# Deficiency in Opu Systems Imparts Salt-Sensitivity to *Weizmannia coagulans*

**DOI:** 10.4014/jmb.2404.04016

**Published:** 2024-06-07

**Authors:** Tao Kim, Sojeong Heo, Jong-Hoon Lee, Do-Won Jeong

**Affiliations:** 1 Department of Food and Nutrition, Dongduk Women’s University, Seoul 02748, Republic of Korea; 2 Pulmuone Institute of Technology, Cheongju 28220, Republic of Korea; 3 Department of Food Science and Biotechnology, Kyonggi University, Suwon 16227, Republic of Korea

**Keywords:** *Weizmannia coagulans*, salt tolerance, *Bacillus*, Opu system, genome

## Abstract

*Weizmannia coagulans* can be used as a starter strain in fermented foods or as a probiotic. However, it is salt-sensitive. Here, *W. coagulans* genomes were compared with the genomes of strains of *Bacillus* species (*B. licheniformis*, *B. siamensis*, *B. subtilis*, and *B. velezensis*) that were isolated from fermented foods and show salt tolerance, to identify the basis for the salt-sensitivity of *W. coagulans*. Osmoprotectant uptake (Opu) systems transport compatible solutes into cells to help them tolerate osmotic stress. *B. siamensis*, *B. subtilis*, and *B. velezensis* each possess five Opu systems (OpuA, OpuB, OpuC, OpuD, and OpuE); *B. licheniformis* has all except OpuB. However, *W. coagulans* only has the OpuC system. Based on these findings, the *opuA* and *opuB* operons, and the *opuD* and *opuE* genes, were amplified from *B. velezensis*. Expression of each of these systems, respectively, in *W. coagulans* increased salt-tolerance. *W. coagulans* expressing *B. velezensis* *opuA*, *opuD*, or *opuE* grew in 10.5% NaCl (w/v), whereas wild-type *W. coagulans* could not grow in 3.5% NaCl. The salt resistance of *B. subtilis* was also increased by overexpression of *B. velezensis* *opuA*, *opuB*, *opuD*, or *opuE*. These results indicate that the salt-susceptibility of *W. coagulans* arises because it is deficient in Opu systems.

## Introduction

*Bacillus coagulans* was first isolated from spoiled evaporated milk [1] and later reclassified as *Weizmannia coagulans* based on genomic analysis [2]. *W. coagulans* is a Gram-positive, lactic acid-producing, facultative anaerobic, spore-forming bacterium. It is able to endure extreme conditions such as high-temperature, food processing, stomach acids, bile, and high salt, in the form of spores. These properties are great advantages for a probiotic strain that must go through the gastrointestinal tract and an industrial strain that must go through food processing steps [3].

*W. coagulans* has been studied for a long time as a probiotic bacterium for humans [4-6]. It has several health benefits, including prevention of muscle damage during exercise, improvement of gastrointestinal disorders, ease of diarrhea, and prevention of bacterial vaginosis [4-6]. Health benefits of *W. coagulans* have been demonstrated in human studies [7, 8], and *W. coagulans* is sold commercially as a probiotic.

*W. coagulans* is naturally distributed in a variety of niches including soil, water, and air. It is also found in food materials such as soybean, locust bean, maize, rice, and others [3, 9-11]. *W. coagulans* produces exo-enzymes such as amylase, protease, and lipase. These activities contribute to improvement of the quality and sensory properties of fermented foods via protein and carbohydrate degradation [3, 12, 13]. *W. coagulans* also produces compounds with activity against Gram-positive and Gram-negative bacteria [14]. Therefore, *W. coagulans* is used in various commercial food products, including dairy foods, fermented meats, cereals, baby foods, and ice cream [3].

The Qualified Presumption of Safety (QPS) system of the European Union Food Safety Authority was introduced to assess the safety of food and feed microorganisms [15], and *W. coagulans* has been on the QPS list since 2007. Also, four *W. coagulans* strains have been given Generally Recognized as Safe status by the US Food and Drug Administration (http://www.fda.gov/) [16]. *W. coagulans* is also on the Food Materials list of the Ministry of Food and Drug Safety, Korea (Notification of the Ministry of Food and Drug Safety No. 2024-20). These classifications confirm that *W. coagulans* is nonpathogenic and can be applied in the food industry, including as a probiotic.

Most research on *W. coagulans* has focused on its functional properties as a probiotic strain, while its use as a starter candidate for fermented foods has been studied less. However, 31 *W. coagulans* strains from rice straw were assessed for their antibiotic susceptibility using the minimum inhibitory concentration test and their enzymatic (protease and lipase) activities, to select safe starter candidates for sensory improvement of fermented foods [17]. At the same time, it was found that *W. coagulans* did not grow on tryptic soy agar (TSA) containing 3% NaCl (w/v). The NaCl concentration of most fermented foods is >2%, and for starters to work efficiently in fermented foods, growth must be possible in the salt concentration of the food. Here, we determined the reason for the salt-sensitivity of *W. coagulans*.

## Materials and Methods

### Bacterial Strains and Culture Conditions

The strains and plasmids used in this study are listed in Table 1. *Escherichia coli* was cultured in Luria-Bertani agar (Becton, Dickinson and Co., USA), *Bacillus* in tryptic soy agar (TSA; Becton, Dickinson and Co.), and *W. coagulans* in de Man, Rogosa, and Sharpe (Becton, Dickinson and Co.) agar. When necessary, antibiotics were added to the growth medium at the following concentrations: ampicillin, 100 μg/ml; erythromycin, 10 μg/ml; kanamycin, 10 μg/ml.

### Comparative Genomic Analyses

For comparative genomic analysis of *W. coagulans* and four *Bacillus* species, which were mainly isolated from fermented foods, genome sequence data were obtained from the NCBI (http://ncbi.nlm.nih.gov/genomes)(Table 2). Genes were predicted using the RAST server for SEED-based automated annotation [40]. The predicted genes of the strains were confirmed using CLgenomics ver. 1.55 software (CJ Bioscience, Republic of Korea) and the iPath (ver. 3) module [41].

### DNA Cloning and Transformation

Plasmids and genomic DNA of *E. coli*, *B. subtilis*, and *W. coagulans* were extracted with an Inclone Plasmid Mini Prep Kit (Inclone Biotech, Republic of Korea) and a DNeasy Tissue Kit (Qiagen, Germany), respectively, according to the manufacturers’ instructions. *B. subtilis* and *W. coagulans* were treated with lysozyme (0.1 g/ml) at 37°C for 1h before DNA extraction.

Genes related to the Opu system from *B. velezensis* DMB07 were amplified by PCR using primer sets containing the appropriate restriction enzyme sites for insertion into pLipSM or pYJ335 (Table 3). PCR amplifications were performed using a T3000 thermocycler (Biometra, Germany) [42] and an Inclone *Taq* Polymerase Kit (Inclone Biotech) according to the manual. PCR primers were designed based on Opu system genes and nearby sequences of *B. velezensis* strain DMB07 (Table 3). All PCRs were performed using 30 cycles of denaturing at 95°C for 1 min, annealing at 63°C for 1.5 min, and elongation at 72°C for 1.5 min. Amplicons were digested by appropriate enzymes and inserted into same sites of pLipSM or pYJ335. Constructed plasmids were verified by PCR using pLipSM-check-F primer or pYJ335-check-F primer and DNA sequencing. Constructed plasmids derived from pYJ335 were introduced into *E. coli* DH5α (Stratagene, USA) by the method of Hanahan and Meselson [43], and then into *W. coagulans* KCTC 3625^T^ (KCTC; Korean Collection for Type Cultures, South Korea) by electroporation [44] with a gene pulser (Bio-Rad, USA). For introduction into *B. subtilis* ISW1214 of pLipSM-derived plasmids, DNA extracted from *E. coli* BL21 (DE3) (New England Biolabs, USA) was used [20].

### Determination of Salt Tolerance

Salt tolerance of the strains in this study was determined by examining growth on TSA supplemented with up to 14% NaCl (w/v, final concentration). Growth on 0.5% (the NaCl concentration in normal TSA), 3.5%, 7%, 10.5%, and 14% NaCl was determined after incubation for 1, 2, 3, 4, and 5 days. The experiment was performed three times, independently.

## Results and Discussion

### Growth of *W. coagulans* on TSA

**Fig. 1:**
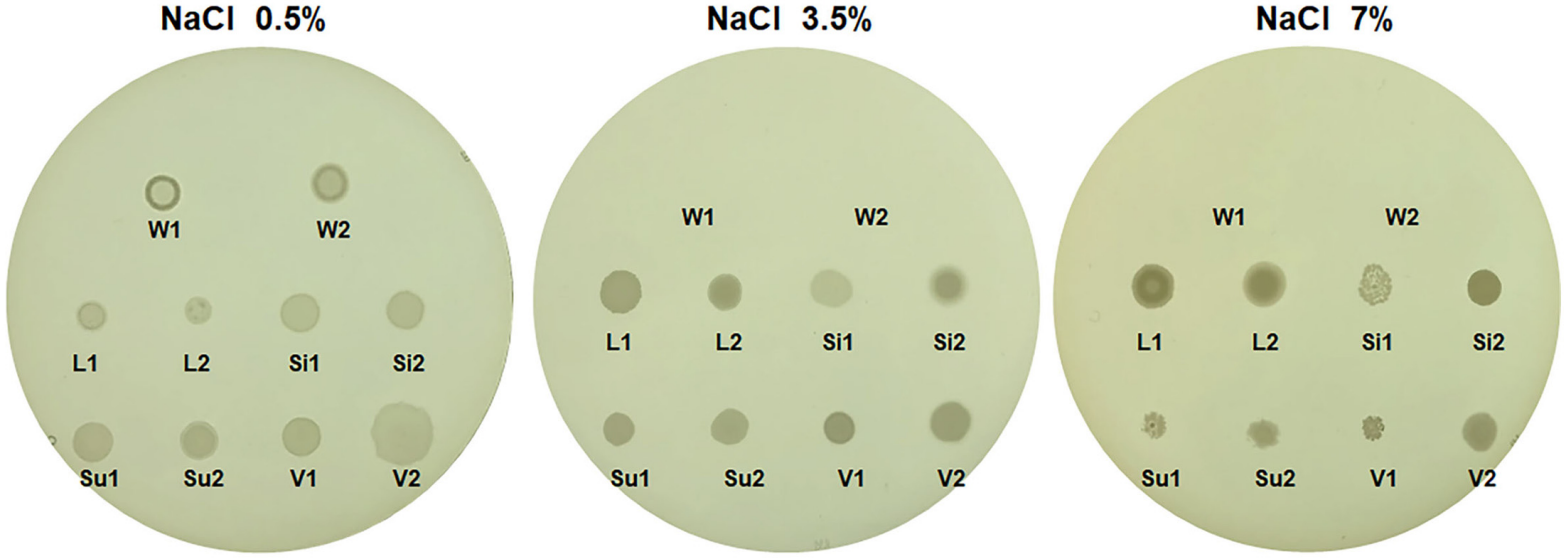
Effect of NaCl on growth of *Weizmannia coagulans* and four *Bacillus* species. Tryptic soy agar (TSA) containing 0%–7% (w/v) NaCl was used for the detection of growth. Strains: W1, *W. coagulans* KCTC 3625^T^; W2, *W. coagulans* ASRS217; L1, *B. licheniformis* KCCM 12145^T^; L2, *B. licheniformis* 0DA23-1; Si1, *B. siamensis* KCTC 13613^T^; Si2, *B. siamensis* B28; Su1, *B. subtilis* KCCM 32835^T^; Su2, *B. subtilis* SRCM102748; V1, *B. velezensis* KCTC 13012^T^; V2, *B. velezensis* DMB07.

*W. coagulans* originally belonged to the genus *Bacillus* but was later reclassified. Most *Bacillus* species can grow on medium supplemented with >7% NaCl (w/v, final concentration) [45-47]. In contrast, *W. coagulans* does not grow at a salt concentration of >3% [17]. To reconfirm this, the growth of *W. coagulans* was checked in different salt conditions. As shown in Fig. 1, *W. coagulans* grew well on TSA without added extra salt (TSA contains 0.5%NaCl), while it did not grow on TSA with final NaCl concentrations of 3.5% or 7%. In contrast, *B. licheniformis*, *B. siamensis*, *B. subtilis*, and *B. velezensis* grew on TSA with a final NaCl concentration of 7% (Fig. 1).

### Comparative Genomic Analysis of Salt Tolerance

*B. licheniformis*, *B. siamensis*, *B. subtilis*, and *B. velezensis* are predominantly isolated from fermented soybean products such as *meju* and *doenjang* [45, 48]. The enzymatic activities of these species contribute to the development of sensory properties of the fermented foods. Therefore, several studies have reported analysis of these strains for use as starters [38, 49, 50]. *W. coagulans* is also isolated from several foods and exhibits protease and lipase activities, so it was judged that it could improve the sensory properties of fermented foods. However, *W. coagulans* is sensitive to salt compared with the four *Bacillus* species. Therefore, we undertook comparative genomic analysis of these species, looking for factors that affect their salt tolerance (Table 2).

**Fig. 2:**
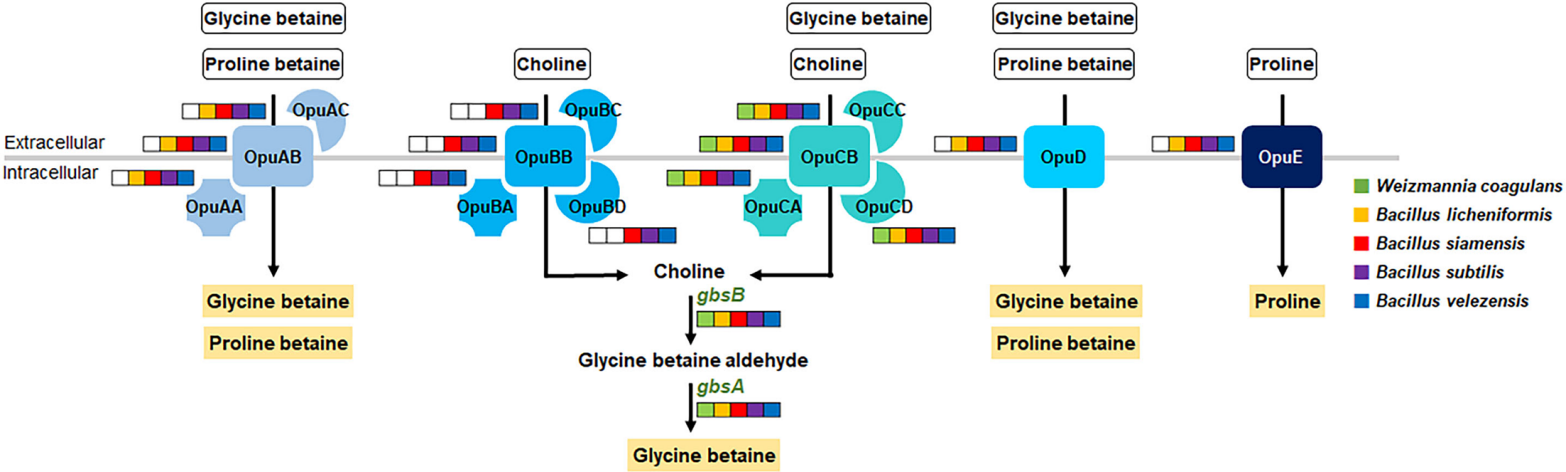
Predicted mechanism of salt-sensitivity of *W. coagulans* based comparative genomic analysis with four *Bacillus* species. Compatible solutes are depicted in black text in yellow boxes. Color coding indicates which species contain which *opu* genes.

To resist osmotic stress, many bacteria accumulate compatible solutes such as choline, glycine betaine, and proline betaine through uptake from outside the cell via transporters including osmoprotectant uptake (Opu) transporter [51, 52]. The Opu system is known to be involved in salt resistance [51]. *W. coagulans* genomes possess the OpuC system. Meanwhile, *B. licheniformis* encodes four Opu systems, and *B. siamensis*, *B. subtilis*, and *B. velezensis* possess five Opu systems (Fig. 2). OpuA, OpuB, and OpuC belong to the ATP-binding cassette transporter superfamily, and OpuD and OpuE are single-component transporters belonging to the BCCT (Betaine/Carnitine/Choline Transporters) family [51]. In the current study, the encoded OpuA, OpuB, and OpuC systems were found to contain the common substrate-binding, transporter, and ATP-binding proteins. Although Hoffmann and Berner reported that *Bacillus* species contain five Opu systems [51], *B. licheniformis* genomes lack the OpuB system. Our results suggest that the low salt-tolerance of *W. coagulans* compared with high salt-tolerance of *Bacillus* species is due to it only having one Opu system.

### Opu Systems Enhance the Salt Tolerance of *B. subtilis*

**Fig. 3:**
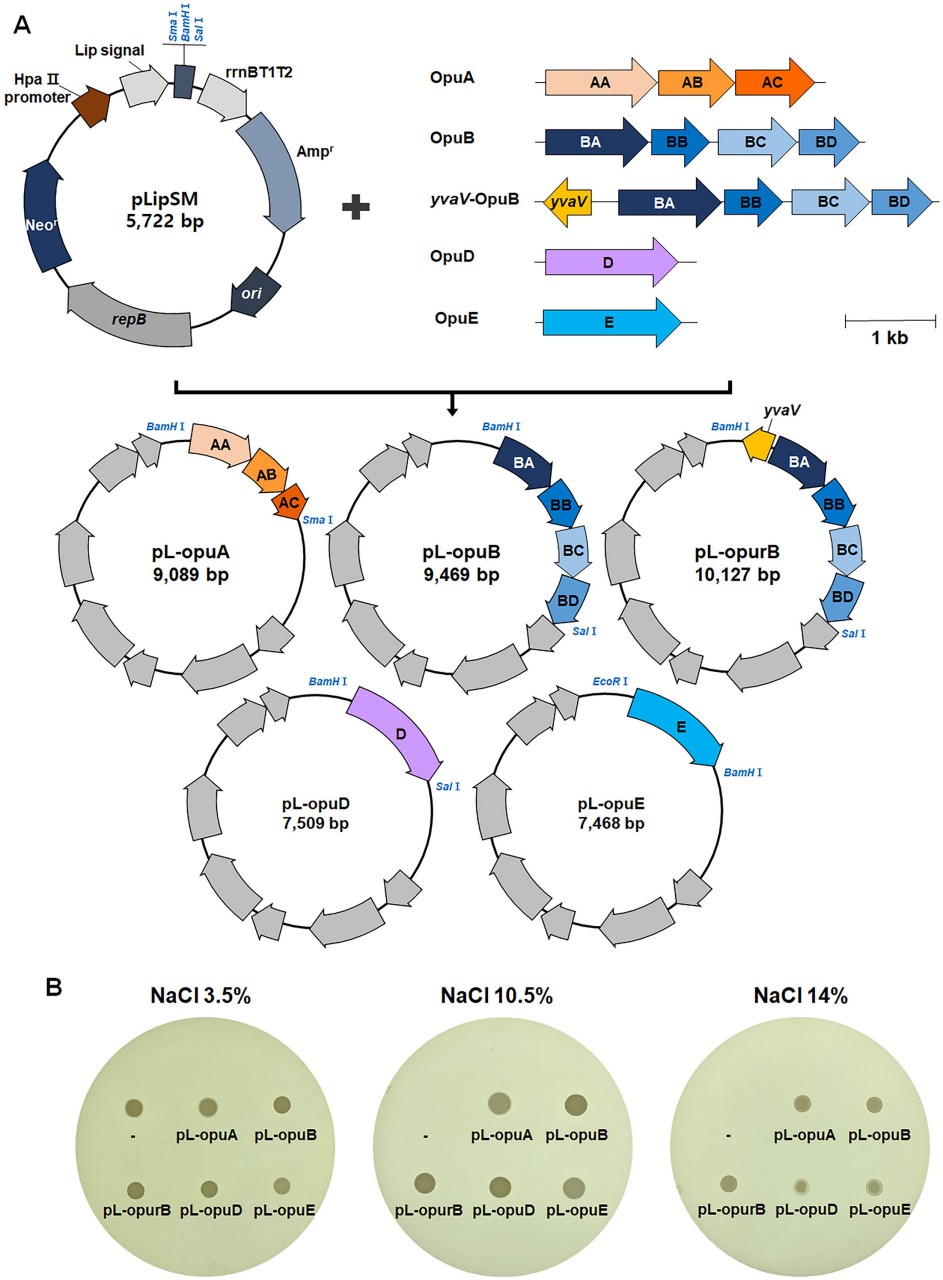
Construction of pLip-SM-derived plasmids containing *opu* genes from *B. velezensis* DMB07 (A) and salt-tolerance of *B. subtilis* containing these plasmids (B). TSA containing up to 14% NaCl (w/v, final concentration) was used for the detection of growth.

As a result of the comparative genome analysis, the salt-sensitivity of *W. coagulans* was reasoned to arise from a deficiency in Opu systems. To test that, we aimed to introduce “absent” genes into *W. coagulans* to determine the effect on salt resistance. Plasmid pLipSM is an *E. coli*/*Bacillus* shuttle vector [20]. Although *W. coagulans* has been reclassified from genus *Bacillus*, this plasmid is also functional in *Weizmannia*. *opu* genes from *B. velezensis* DMB07 (which contains all the *opu* genes and had the highest salt resistance among the strains we tested) were cloned and introduced into *W. coagulans* via this plasmid. Note that we did not clone opuC from *B. velezensis* DMB07 because *W. coagulans* naturally possesses this system (see section 2.2). The obtained recombinant plasmids were designated pL-*opuA*, pL-*opuB*, pL-*opuD*, and pL-*opuE*, respectively. In addition, pL-opurB was reconstructed with the *opuB* operon and the *yvaV* gene, which is a regulator for expression of the *opuB* operon (Fig. 3A).

Because *W. coagulans* strain KCTC 3625^T^ is a wild-type, it was not expected to be easy to introduce recombinant plasmids into the strain, so they were first introduced into laboratory strain *B. subtilis* ISW1214. We tested whether the protein expression vectors had an effect on the salt resistance of transformed *B. subtilis* ISW1214. As shown in Fig. 3B, wild-type *B. subtilis* ISW1214 grew in medium containing 7% NaCl, but not in medium containing 10.5% or 14% NaCl. However, when any one of the *B. velezensis* Opu systems was overexpressed, the resulting strain grew on 14% NaCl. The multicomponent systems OpuA and OpuB enabled better growth on 14%NaCl than the single-component systems OpuD and OpuE (Fig. 3B).

### Opu Systems Confer Salt Tolerance to *W. coagulans*

**Fig. 4:**
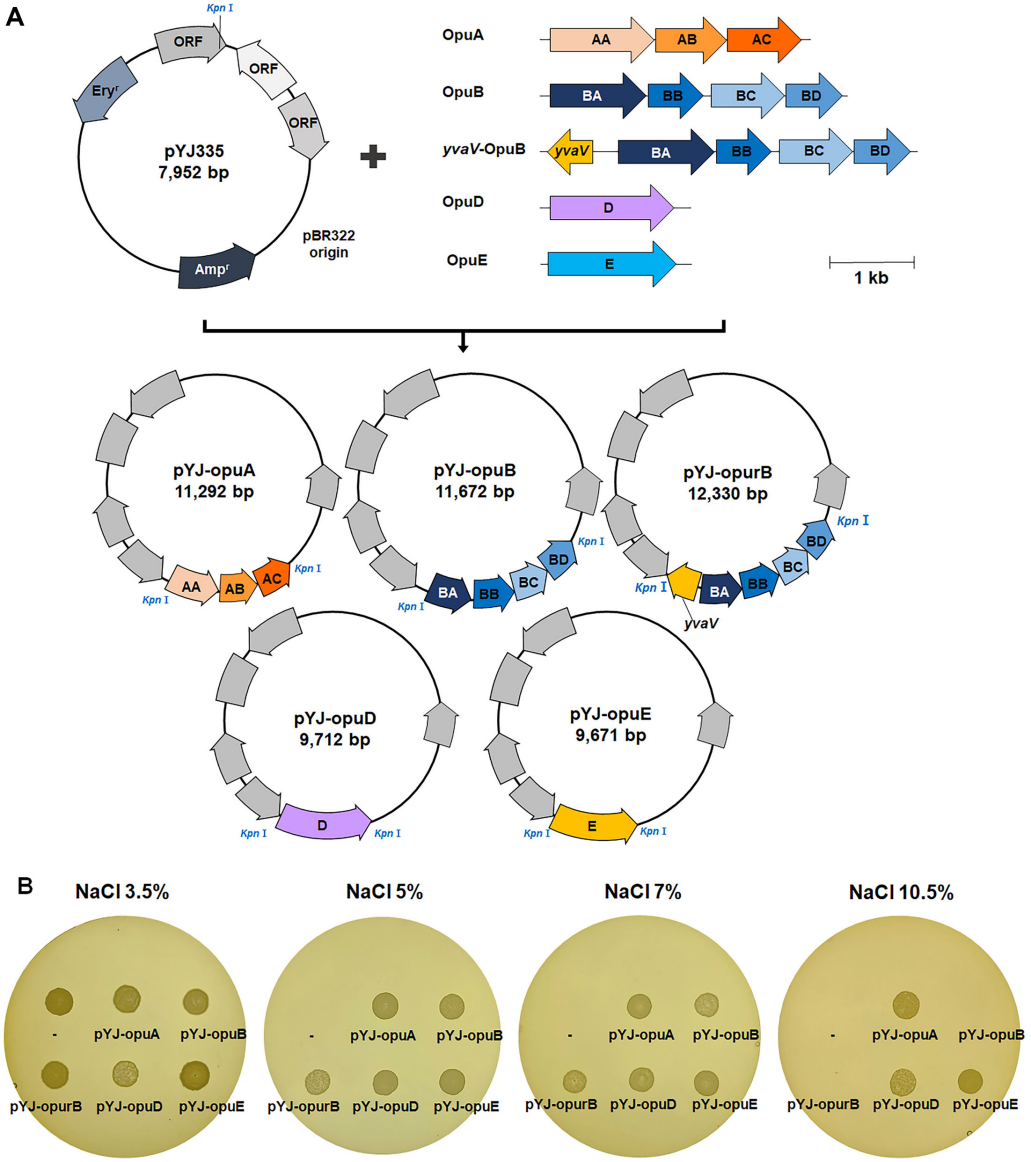
Construction of pYJ335-derived plasmids containing *opu* genes from *B. velezensis* DMB07 (A) and salt-tolerance of *W. coagulans* containing these plasmids (B). de Man, Rogosa, and Sharpe medium containing up to 10.5% NaCl (w/v, final concentration) was used for the detection of growth.

pLipSM-based plasmids produced in *B. subtilis* were purified and introduced into *W. coagulans* KCTC 3625^T^, but *W. coagulans* KCTC 3625^T^ showed resistance to low concentrations of kanamycin, which is the selection marker in pLipSM, so it was not easy to select *W. coagulans* transformants containing pLipSM-derived plasmids. Accordingly, we tried to introduce *opu* genes into *W. coagulans* KCTC 3625^T^ by using pYJ335, which has the erythromycin resistance gene as a selection marker. The replicon of plasmid pYJ335 was derived from pUC19 and pE194 fragments [21]. The replicon can be cloned from lactic acid bacteria and *Staphylococcus* [53]. The *opu* genes were amplified from *B. velezensis* DMB07 in the same way as in the previous experiment (see section 3.3), and recombinant plasmids were generated by inserting the *opuA* and *opuB* operons, and *opuD* and *opuE* genes, to produce pYJ-*opuA*, pYJ-*opuB*, pYJ-*opuD*, and pYJ-*opuE* (Fig. 4A). The vector containing the *opuB* operon, including the *yvaV* gene, which is a regulator for expression of the *opuB* operon, was named pYJ-opurB. These vectors were each transformed directly into *W. coagulans* (without going through *B. subtilis*). All the transformed strains grew in medium with 7% NaCl added, and all except the OpuB-containing transformants grew in 10.5%salt (Fig. 4B).

In previous studies, 67.7% and 71.0% of *W. coagulans* strains showed protease and lipase activities, respectively; these activities produce amino acids and fragrance components from lipids, respectively [17]. In addition, it can be predicted that all the strains of *W. coagulans* have production performance in the condition containing NaCl, and that most strains can inhibit the growth of other microorganisms via antibacterial activity [17] However, the *W. coagulans* strains were sensitive to salt. Most fermented foods contain a significant salt content. Therefore, even though *W. coagulans* is widely distributed in the environment [3, 9-11, 54, 55] and in the raw materials used to prepare fermented foods, detection of this species is lowered in foods with added salt [17]. If *W. coagulans* were to be salt-resistant, it would be expected to play a greater role in fermented food production. Here, the genetic basis of the sensitivity of *W. coagulans* to salt, *i.e.* a deficiency in Opu systems, was determined. These results will help with screening for salt-resistant strains.

## Figures and Tables

**Table 1:** Bacterial strains and plasmids used in this study.

Strain/plasmid	Relevant characteristic(s)	Source or reference
Strain		
*W. coagulans*		
KCTC 3625^T^	*Weizmannia coagulans* type strain, wild-type strain	Korean Collection for Type
		Cultures (KCTC), South Korea
ASRS217	Potential starter candidate, isolated from rice straw	[17]
*B. licheniformis*		
KCCM 12145^T^	*Bacillus licheniformis* type strain	Korean Culture Center of
		Microorganisms (KCCM), South Korea
0DA23-1	Potential starter candidate, isolated from commercial *doenjang*	[18]
*B. siamensis*		
KCTC 13613^T^	*B. siamensis* type strain	KCTC, South Korea
B28	Potential starter candidate, isolated from *kimchi*	[19]
*B. subtilis*		
KCCM 32835^T^	*B. subtilis* type strain	KCCM, South Korea
SRCM102748	Isolated from *kimchi*	SRCM, South Korea
ISW1214	*hsrM1, leuA8, metB5, Tet^5^*	Takara Bio, Japan
*B. velezensis*		
KCTC 13012^T^	*B. velezensis* type strain	KCTC, South Korea
DMB07	Isolated from fermented soybean	Unpublished results
*E. coli*		
DH5α	*Escherichia coli*, cloning host for recombinant plasmids	Stratagene, USA
BL21 (DE3)	*E. coli* *recA*+ strain, host for protein expression	NEB, USA
Plasmid		
pLipSM	*E. coli*–*Bacillus* shuttle vector, cloning vector, Amp^r^, Kan^r^	[20]
pL-opuA	pLipSM derivative containing *opuA* operon	This study
pL-opuB	pLipSM derivative containing *opuB* operon	This study
pL-opurB	pLipSM derivative containing *yvaV* and *opuB* operon	This study
pL-opuD	pLipSM derivative containing *opuD*	This study
pL-opuE	pLipSM derivative containing *opuE*	This study
pYJ335	*E. coli*–staphylococcal shuttle vector, Amp^r^, Ery^r^	[21]
pYJ-opuA	pYJ335 derivative containing *opuA* operon	This study
pYJ-opuB	pYJ335 derivative containing *opuB* operon	This study
pYJ-opurB	pYJ335 derivative containing *yvaV* and *opuB* operon	This study
pYJ-opuD	pYJ335 derivative containing *opuD*	This study
pYJ-opuE	pYJ335 derivative containing *opuE*	This study

**Table 2:** Genomic features of strains of *Weizmannia coagulans* and four *Bacillus* species.

Species	Strain	Size (bp)	G+C content (mol%)	Origin	Country	Accession no.	Reference
*W. coagulans*	KCTC 3625^T^	3,366,995	46.90	Dairy (evaporated milk)	USA	NZ_CP009709	[22]
	ASRS217	3,514,330	46.47	Rice straw	South Korea	NZ_CP058594	[23]
	HM-08	3,624,641	46.30	Healthy chicken intestine	China	NZ_CP010525	[24]
	IDCC1201	3,664,215	46.20	Green malt	South Korea	NZ_CP035305	[9]
	DSM 2314	3,628,651	46.24	Rhizosphere	unknown	NZ_CP033687	[25]
*B. licheniformis*	KCCM 12145^T^	4,222,597	46.20	Unknown	unknown	NZ_CP034569	[26]
	0DA23-1	4,405,373	46.00	Doenjang	South Korea	NZ_CP031126	[27]
	14ADL4	4,332,232	45.90	Doenjang	South Korea	NZ_CP026673	[18]
	MCC 2514	4,230,480	46.20	Raw milk (sheep)	India	NZ_CP038186	[28]
	TCCC 11148	4,341,076	45.90	Soil	unknown	NZ_CP033218	[29]
*B. siamensis*	KCTC 13613^T^	3,779,696	46.30	Salted crab	South Korea	AJVF01000000-51	[30]
	B28	3,946,178	45.89	Kimchi	South Korea	NZ_CP066219-21	[19]
	SCSIO 05746	4,268,316	45.98	Sea mud	Indian Ocean	NZ_CP025001	[31]
*B. subtilis*	KCCM 32835T	4,215,607	43.34	Soil under a mango tree	unknown	NZ_CP020102-3	[32]
	SRCM102748	4,210,797	43.60	Kimchi	South Korea	NZ_CP028212	[33]
	PS832	4,215,367	43.50	Soil	unknown	NZ_CP010053	[34]
	HRBS-10TDI13	4,186,269	43.29	Soybean paste	South Korea	NZ_CP015222	-
	GFR-12	4,202,955	43.30	Chung-gook-jang	South Korea	NZ_CP032852	[35]
*B. velezensis*	KCTC 13012T	4,034,335	46.30	River Velez	Spain	NZ_LLZC01000001-24	[36]
	DMB05	3,262,563	46.25	Meju	South Korea	NZ_CP083715-7	[37]
	DMB06	4,157,945	46.20	Doenjang	South Korea	NZ_CP083763	[38]
	DMB07	4,157,945	45.60	Meju	South Korea	NZ_CP083764	-
	KMU01	3,932,437	46.50	Kimchi	South Korea	NZ_CP063768	[39]

- mean that there are no papers published

**Table 3:** Oligonucleotides used in this study.

Oligonucleotide	Sequence (5 → 3)^a^	Use	Amplified size (bp)
plipSM vector			
*opuAA*-*Bam*HI-F’	CGGGATCCGCCTGATAAAAGCCCGGTTTCC	*opuAA* upstream	3,367
*opuAC*-*Sma*I-R’	TCCCCCGGGGGATGAACCTCTTGTGACAACC	*opuAC* downstream	
*opuBA*-*Bam*HI-F’	CGCGTCGACGCTCATTTGATTACCCCTCTGC	*opuBA* upstream	3,747
*opuBD*-*Sal*I-R’	CGGGATCCCCGGTCAATACGGGTAAATC	*opuBD* downstream	
*yvaV*-*Bam*HI-F’	CGGGATCCGAAAAAACGAACCAAAGCGCCG	*yvaV* downstream	4,405
*opuD*-*Bam*HI-F’	CGGGATCCCGTCCCCGTTGATAATTGACC	*opuD* upstream	1,787
*opuD*-*Sal*I-R’	ACGCGTCGACCCTGTGATCCTGAAGGTGAGC	*opuD* downstream	
*opuE*-*Eco*RI-F’	CGCAATTCGGTTTAGTAACCATAGCCGGC	*opuE* upstream	1,746
*opuE*-*Bam*HI-R’	CGGGATCCGCTCAATTTGCACAGCACCTCC	*opuE* downstream	
plipSM-check-F’	CCAGCCGAAAGAAGCCAAAGC	Hpa II promoter downstream, upstream of insertion site	
pYJ335 vector			
*opuAA*-*Kpn*I-F’	CCGGTACCGCCTGATAAAAGCCCGGTTTCC	*opuAA* upstream	3,367
*opuAC*-*Kpn*I-R’	CCGGTACCGGATGAACCTCTTGTGACAACC	*opuAA* downstream	
*opuBA*-*Kpn*I-F’	CCGGTACCGCTCATTTGATTACCCCTCTGC	*opuBA* upstream	3,747
*opuBD*-*Kpn*I-R’	CCGGTACCCCGGTCAATACGGGTAAATC	*opuBD* downstream	
*yvaV*-*Kpn*I-F’	CCGGTACCGAAAAAACGAACCAAAGCGCCG	*yvaV* downstream	4,405
*opuD*-*Kpn*I-F’	CCGGTACCCGTCCCCGTTGATAATTGACC	*opuD* upstream	1,787
*opuD*-*Kpn*I-R’	CCGGTACCCCTGTGATCCTGAAGGTGAGC	*opuD* downstream	
*opuE*-*Kpn*I-F’	CCGGTACCGGTTTAGTAACCATAGCCGGC	*opuE* upstream	1,746
*opuE*-*Kpn*I-R’	CCGGTACCGCTCAATTTGCACAGCACCTCC	*opuE* downstream	
pYJ335-check-F’	GCGATTAAGTTGGGTAACGC	*Kpn*I site upstream of pYJ335	

^a^Restriction sites are underline.

## References

[1] Hammer BW (1915). Bacteriological studies on the coagulation of evaporated milk (Vol. 17).

[2] Gupta RS, Patel S, Saini N, Chen S (2020). Robust demarcation of 17 distinct *Bacillus* species clades, proposed as novel *Bacillaceae* genera, by phylogenomics and comparative genomic analyses: description of *Robertmurraya kyonggiensis* sp. nov. and proposal for an emended genus *Bacillus* limiting it only to the members of the *Subtilis* and *Cereus* clades of species. Int. J. Syst. Evol. Microbiol..

[3] Konuray G, Erginkaya Z (2018). Potential use of *Bacillus coagulans* in the food industry. Foods.

[4] Jäger R, Shields KA, Lowery RP, De Souza EO, Partl JM, Hollmer C (2016). Probiotic *Bacillus coagulans* GBI-30, 6086 reduces exercise-induced muscle damage and increases recovery. Peer J..

[5] Majeed M, Majeed S, Nagabhushanam K, Natarajan S, Sivakumar A, Ali F (2016). Evaluation of the stability of *Bacillus coagulans* MTCC 5856 during processing and storage of functional foods. Int. J. Food Sci. Technol..

[6] Ratna Sudha, M Yelikar KA, Deshpande S (2012). Clinical study of *Bacillus coagulans* unique IS-2 (ATCC PTA-11748) in the treatment of patients with bacterial vaginosis. Indian J. Microbiol..

[7] Elshaghabee FMF, Rokana N, Gulhane RD, Sharma C, Panwar H (2017). Bacillus as potential probiotics: status, concerns, and future perspectives. Front. Microbiol..

[8] Maity C, Gupta AK, Saroj DB, Biyani A, Bagkar P, Kulkarni J (2021). Impact of a gastrointestinal stable probiotic supplement *Bacillus coagulans* LBSC on human gut microbiome modulation. J. Diet. Suppl..

[9] Bang WY, Ban OH, Lee BS, Oh S, Park C, Park MK (2021). Genomic-, phenotypic-, and toxicity-based safety assessment and probiotic potency of *Bacillus coagulans* IDCC 1201 isolated from green malt. J. Ind. Microbiol. Biotechnol..

[10] Lee B, Lee H, Jeong DW, Lee JH (2015). A rapid isolation method for *Bacillus coagulans* from rice straw. Microbiol. Biotechnol. Lett..

[11] Shudong P, Guo C, Wu S, Cui H, Suo H, Duan Z (2022). Bioactivity and metabolomics changes of plant-based drink fermented by *Bacillus coagulans* VHProbi C08. LWT - Food Sci. Technol..

[12] Babu KR, Satyanarayana T (1995). α-Amylase production by thermophilic *Bacillus coagulans* in solid state fermentation. Process Biochem..

[13] Olajuyigbe FM, Ehiosun KI (2013). Production of thermostable and organic solvent-tolerant alkaline protease from *Bacillus coagulans* PSB-07 under different submerged fermentation conditions. Afr. J. Biotechnol..

[14] Le Marrec C, Hyronimus B, Bressollier P, Verneuil B, Urdaci MC (2000). Biochemical and genetic characterization of coagulin, a new antilisterial bacteriocin in the pediocin family of bacteriocins, produced by *Bacillus coagulans* I4. Appl. Environ. Microbiol..

[15] EFSA (2007). Introduction of a qualified presumption of safety (QPS) approach for assessment of selected microorganisms referred to EFSA. EFSA J..

[16] Heo S, Kim T, Na HE, Lee G, Park JH, Park HJ Safety assessment systems for microbial starters derived from fermented foods. J. Microbiol. Biotechnol..

[17] Kim YS, Lee J, Heo S, Lee JH, Jeong DW (2021). Technology and safety evaluation of *Bacillus coagulans* exhibiting antimicrobial activity for starter development. LWT - Food Sci. Technol..

[18] Jeong DW, Lee B, Lee J H (2018). Complete genome sequence of *Bacillus licheniformis* 14ADL4 exhibiting resistance to clindamycin. Korean J. Microbiol..

[19] Heo S, Kim JH, Kwak MS, Jeong DW, Sung MH (2021). Functional genomic insights into probiotic *Bacillus siamensis* strain B28 from traditional Korean fermented kimchi. Foods.

[20] Lee MH, Song JJ, Choi YH, Hong SP, Rha E, Kim HK (2003). High-level expression and secretion of *Bacillus pumilus* lipase B26 in *Bacillus subtilis* Chungkookjang. J. Microbiol. Biotechnol..

[21] Ji Y, Marra A, Rosenberg M, Woodnutt G (1999). Regulated antisense RNA eliminates alpha-toxin virulence in *Staphylococcus aureus* infection. J. Bacteriol..

[22] Johnson SL, Bishop-Lilly KA, Ladner JT, Daligault HE, Davenport KW, Jaissle J (2015). Complete genome sequences for 59 *Burkholderia* isolates, both pathogenic and near neighbor. Genome Announc..

[23] Lee J, Heo S, Kim Y S, Lee JH, Jeong DW (2020). Complete genome sequence of *Bacillus coagulans* strain ASRS217, a potential food fermentation starter culture. Korean J. Microbiol..

[24] Yao G, Gao P, Zhang W (2016). Complete genome sequence of probiotic *Bacillus coagulans* HM-08: a potential lactic acid producer. J. Biotechnol..

[25] Maas RH, Bakker RR, Jansen ML, Visser D, Jong E, Eggink G (2008). Lactic acid production from lime-treated wheat straw by *Bacillus coagulans*: neutralization of acid by fed-batch addition of alkaline substrate. Appl. Microbiol. Biotechnol..

[26] Rey MW, Ramaiya P, Nelson BA, Brody-Karpin SD, Zaretsky EJ, Tang M (2004). Complete genome sequence of the industrial bacterium *Bacillus licheniformis* and comparisons with closely related *Bacillus* species. Genome Biol..

[27] Jeong DW, Lee B, Heo S, Jang M, Lee JH (2018). Complete genome sequence of *Bacillus licheniformis* strain 0DA23-1, a potential starter culture candidate for soybean fermentation. Korean J. Microbiol..

[28] Rohith HS, Halami PM (2021). The combined effect of potential probiotic *Bacillus licheniformis* MCC 2514 and *Bifidobacterium breve* NCIM 5671 towards anti-inflammatory activity on HT-29 cell lines. Probiotics Antimicrob. Proteins.

[29] Yuan F, Li K, Zhou C, Zhou H, Liu H, Chai H (2020). Identification of two novel highly inducible promoters from *Bacillus licheniformis* by screening transcriptomic data. Genom.

[30] Jeong H, Jeong DE, Kim SH, Song GC, Park SY, Ryu CM (2012). Draft genome sequence of the plant growth-promoting bacterium *Bacillus siamensis* KCTC 13613^T^. J. Bacteriol..

[31] Pan H, Tian X, Shao M, Xie Y, Huang H, Hu J (2019). Genome mining and metabolic profiling illuminate the chemistry driving diverse biological activities of *Bacillus siamensis* SCSIO 05746. Appl. Microbiol. Biotechnol..

[32] Nye TM, Schroeder JW, Kearns DB, Simmons LA (2017). Complete genome sequence of undomesticated *Bacillus subtilis* strain NCIB 3610. Genome Announc..

[33] Iqbal S, Ullah N, Janjua HA (2021). *In vitro* evaluation and genome mining of *Bacillus subtilis* strain RS10 reveals its biocontrol and plant growth-promoting potential. Agriculture.

[34] Grela A, Jamrozek I, Hubisz M, Iwanicki A, Hinc K, Kazmierkiewicz R (2018). Positions 299 and 302 of the GerAA subunit are important for function of the GerA spore germination receptor in *Bacillus subtilis*. PLoS One.

[35] Heo J, Kim JS, Hong SB, Park BY, Kim SJ, Kwon SW (2019). Genetic marker gene, *recQ*, differentiating *Bacillus subtilis* and the closely related *Bacillus* species. FEMS Microbiol. Lett..

[36] Dunlap CA, Kim SJ, Kwon SW, Rooney AP (2016). *Bacillus velezensis* is not a later heterotypic synonym of *Bacillus amyloliquefaciens*; *Bacillus methylotrophicus*, *Bacillus amyloliquefaciens* subsp. *plantarum* and '*Bacillus oryzicola*' are laterheterotypic synonyms of *Bacillus velezensis* based on phylogenomics. Int. J. Syst. Evol. Microbiol..

[37] Na HE, Heo S, Kim T, Lee G, Lee JH, Jeong DW (2023). ComQXPA quorum-sensing systems contribute to enhancing the protease activity of *Bacillus velezensis* DMB05 from fermented soybeans. Int. J. Food Microbiol..

[38] Na HE, Heo S, Kim YS, Kim T, Lee G, Lee JH (2022). The safety and technological properties of *Bacillus velezensis* DMB06 used as a starter candidate were evaluated by genome analysis. LWT - Food Sci. Technol..

[39] Heo S, Kim JH, Kwak MS, Sung MH, Jeong DW (2021). Functional annotation genome unravels potential probiotic *Bacillus velezensis* strain KMU01 from traditional Korean fermented kimchi. Foods.

[40] Aziz RK, Bartels D, Best AA, DeJongh M, Disz T, Edwards RA (2008). The RAST Server: rapid annotations using subsystems technology. BMC Genom..

[41] Darzi Y, Letunic I, Bork P, Yamada T (2018). iPath3.0: interactive pathways explorer v3. Nucleic Acids Res..

[42] Abbas BA, Khudor MH, Saeed BMS (2014). Detection of *hbl*, *nhe* and *bceT* toxin genes in *Bacillus cereus* isolates by multiplex PCR. Int. J. Curr. Microbiol. Appl. Sci..

[43] Hanahan D, Meselson M (1983). Plasmid screening at high colony density. Methods Enzymol..

[44] Kraemer GR, Iandolo JJ (1990). High-frequency transformation of *Staphylococcus aureus* by electroporation. Curr. Microbiol..

[45] Jeong DW, Kim HR, Jung G, Han S, Kim CT, Lee JH (2014). Bacterial community migration in the ripening of doenjang, a traditional Korean fermented soybean food. J. Microbiol. Biotechnol..

[46] Jeong DW, Jeong M, Lee JH (2017). Antibiotic susceptibilities and characteristics of *Bacillus licheniformis* isolates from traditional Korean fermented soybean foods. LWT - Food Sci. Technol..

[47] Kim T, Heo S, Na HE, Lee G, Kim JH, Kwak MS (2022). Bacterial community of galchi-baechu kimchi based on culturedependent and -independent investigation and selection of starter candidates. J. Microbiol. Biotechnol..

[48] Kim YS, Kim MC, Kwon SW, Kim SJ, Park IC, Ka JO (2011). Analyses of bacterial communities in meju, a Korean traditional fermented soybean bricks, by cultivation-based and pyrosequencing methods. J. Microbiol. Biotechnol..

[49] Jeon HH, Jung JY, Chun BH, Kim MD, Baek SY, Moon JY (2016). Screening and characterization of potential *Bacillus* starter cultures for fermenting low-salt soybean paste (Doenjang). J. Microbiol. Biotechnol..

[50] Lee MY, Park SY, Jung KO, Park KY, Kim SD (2005). Quality and functional characteristics of chungkukjang prepared with various *Bacillus* sp. isolated from traditional chungkukjang. J. Food Sci..

[51] Hoffmann T, Bremer E (2017). Guardians in a stressful world: the Opu family of compatible solute transporters from *Bacillus subtilis*. Biol. Chem..

[52] Heo S, Lee J, Lee JH, Jeong DW (2019). Genomic insight into the salt tolerance of *Enterococcus faecium*, *Enterococcus faecalis* and *Tetragenococcus halophilus*. J. Microbiol. Biotechnol..

[53] Peterbauer C, Maischberger T, Haltrich D (2011). Food-grade gene expression in lactic acid bacteria. Biotechnol. J..

[54] Wang L, Xue Z, Zhao B, Yu B, Xu P, Ma Y (2013). Jerusalem artichoke powder: a useful material in producing high-optical-purity Llactate using an efficient sugar-utilizing thermophilic *Bacillus coagulans* strain. Bioresour. Technol..

[55] Xing SC, Chen JY, Lv N, Mi JD, Chen WL, Liang JB (2018). Biosorption of lead (Pb2+) by the vegetative and decay cells and spores of *Bacillus coagulans* R11 isolated from lead mine soil. Chemosphere.

